# BRIP1 (BACH1) variants and familial breast cancer risk: a case-control study

**DOI:** 10.1186/1471-2407-7-83

**Published:** 2007-05-15

**Authors:** Bernd Frank, Kari Hemminki, Alfons Meindl, Barbara Wappenschmidt, Christian Sutter, Marion Kiechle, Peter Bugert, Rita K Schmutzler, Claus R Bartram, Barbara Burwinkel

**Affiliations:** 1 Helmholtz-University Group Molecular Epidemiology, German Cancer Research Center, DKFZ, Heidelberg, Germany; 2 Division of Molecular Genetic Epidemiology, German Cancer Research Center, DKFZ, Heidelberg, Germany; 3 Center for Family Medicine, Karolinska Institute, Huddinge, Sweden; 4 Department of Gynaecology and Obstetrics, Klinikum rechts der Isar at the Technical University, Munich, Germany; 5 Division of Molecular Gynaeco-Oncology, Department of Gynaecology and Obstetrics, Clinical Center University of Cologne, Germany; 6 Center of Molecular Medicine Cologne (CMMC), University Hospital of Cologne, Germany; 7 Institute of Human Genetics, University of Heidelberg, Heidelberg, Germany; 8 Institute of Transfusion Medicine and Immunology, Red Cross Blood Service of Baden-Württemberg-Hessia, University of Heidelberg, Faculty of Clinical Medicine, Mannheim, Germany

## Abstract

**Background:**

Inactivating and truncating mutations of the nuclear BRCA1-interacting protein 1 (BRIP1) have been shown to be the major cause of Fanconi anaemia and, due to subsequent alterations of BRCA1 function, predispose to breast cancer (BC).

**Methods:**

We investigated the effect of BRIP1 -64G>A and Pro919Ser on familial BC risk by means of TaqMan allelic discrimination, analysing *BRCA1/BRCA2 *mutation-negative index patients of 571 German BC families and 712 control individuals.

**Results:**

No significant differences in genotype frequencies between BC cases and controls for BRIP1 -64G>A and Pro919Ser were observed.

**Conclusion:**

We found no effect of the putatively functional BRIP1 variants -64G>A and Pro919Ser on the risk of familial BC.

## Background

Germline mutations in the high-penetrance genes *BRCA1 *and *BRCA2 *account for up to 25% of the hereditary forms of breast cancer (BC) [1,2]. The nuclear BRCA1-interacting protein 1 (BRIP1; also referred to as BRCA1-associated C-terminal helicase, BACH1) directly binds the BRCT-motif containing domain of BRCA1, thus likely contributing to its DNA repair and tumour suppressor functions [3,4]. *BRIP1 *deficiency has been described as the causation for cancer-predisposing Fanconi anaemia [5,6], and a recent study has identified constitutional truncating *BRIP1 *mutations to confer susceptibility to BC [7].

Several genotyping studies have addressed the association between BRIP1 variants and BC risk, but the results have remained controversial [8-11]. The non-conservative BRIP1 Pro919Ser substitition has been previously reported to be associated with an increased BC risk up to age 50 [12]. We evaluated the effects of BRIP -64G>A, which may affect gene regulation, and Pro919Ser, on a large German familial BC study cohort.

## Methods

### Study population

The familial breast cancer cohort comprised 571 unrelated German, female index cases (19 to 87 years of age; median 45) without deleterious *BRCA1 *and *BRCA2 *mutations. They were collected during the years 1996–2005 through the Institute of Human Genetics (Heidelberg, Germany), the Department of Gynaecology and Obstetrics (Cologne, Germany) and the Department of Medical Genetics (Munich, Germany). According to the German Consortium for Hereditary Breast and Ovarian Cancer, breast cancer cases are classified into six categories based on family history: (A1) families with two or more breast cancer cases including at least two cases with onset below the age of 50 years; (A2) families with at least one male breast cancer case; (B) families with at least one breast cancer and one ovarian cancer case; (C) families with at least two breast cancer cases including one case diagnosed before the age of 50 years; (D) families with at least two breast cancer cases diagnosed after the age of 50 years; (E) single cases of breast cancer with age of diagnosis before 35 years [2]. All women gave written consent to the molecular analysis of the *BRCA1 *and *BRCA2 *genes and potential new breast cancer susceptibility genes. Prior to DNA extraction, their blood (EDTA) was frozen at -20°C. The DNA was isolated by a conventional phenol-chloroform protocol. Mutations in the open reading frame of *BRCA1 *and *BRCA2 *were excluded by applying denaturing high performance liquid chromatography (DHPLC) on all exons, followed by direct sequencing of conspicuous exons. The control series consisted of 712 healthy, unrelated female blood donors (26 to 68 years of age, median 49). They were recruited in 2004 and 2005 by the Institute of Transfusion Medicine and Immunology (Mannheim, Germany) and share the ethnic background with the breast cancer patients. According to the German guidelines for blood donation, all blood donors were examined by a standard questionnaire. Buffy coat samples were taken from the anti-coagulated blood donations and were used for DNA isolation (FlexiGene^® ^DNA Kit; Qiagen, Hilden Germany). All blood donors consented to the use of their samples for research studies. The study was approved by the Ethics Committee of the University of Heidelberg (Heidelberg, Germany).

### Genotyping

*BRIP1 *genotyping was done by the TaqMan allelic discrimination method as previously described [13]. TaqMan primers and probes were provided by the assay-by-design service (Applied Biosystems, Foster City, CA) and designed on the basis of the GenBank NT_010783 sequence. Sequences of primers and probes are available upon request.

### Statistical analysis

Genotype-specific odds ratios (ORs), 95% confidence intervals (95% CIs) and *P *values were computed by unconditional logistic regression using the Statistical Analysis System software (Version 9.1.; SAS Institute Inc., Cary, NC). Haplotypes were inferred using the SNPHAP programme created by D. Clayton [14]. Power calculation was carried out with the power and sample size calculation software PS version 2.1.31 [15].

### Transcription factor search

The search for putative transcription factors was performed using TESS (Transcription Element Search Software, [16]) and TFSEARCH (Searching Transcription Factor Binding Sites, [17]).

## Results and discussion

Mutations in the DNA helicase *BRIP1 *have been implicated in the aetiology of Fanconi anaemia, a genetic disorder that is characterised by congenital abnormalities, progressive bone marrow failure, genomic instability and predisposition to cancer [5-7]. Our study assessed the relevance of the BRIP1 variants -64G>A and Pro919Ser to familial BC. According to transcription factor binding site searches, the replacement of G by A at position -64 leads to the formation of a GATA or CCAAT motif, suggesting an modification of gene expression. BRIP Pro919Ser is located in the BRCA1-interacting domain and may alter protein structure and function.

Genotype frequencies for the analysed polymorphisms were in agreement with Hardy-Weinberg expectations in controls. No significant differences in genotype frequencies between BC cases and controls for either BRIP1 -64G>A or Pro919Ser were observed (see Table 1). Adjustment for age made no significant difference to the results, hence only unadjusted ORs are presented. Our findings are in accord with previously published data [8,11]. Though a recent kin-cohort study has shown a strong association with 4.5- to 6.9-fold familial BC risk for Pro919Ser in premenopausal women [12], our data do not support the observed effect when stratified according to age at diagnosis (see Table 1). Haplotype analysis with BRIP1 -64G>A and Pro919Ser did not indicate any association with familial BC risk (data not shown).

The strengths of the present study are represented by a sound sample size and a homogeneous study cohort of a single ethnic group, comprising women selected for familial BC. Only *BRCA1 *and *BRCA2 *mutation-negative familial BC cases were considered in order to avoid effects caused by these high-penetrance susceptibility genes. With the present sample size, we had a power of 80% at a significance level of 0.05 to detect an OR of ≥ 1.44 for both -64G>A and Pro919Ser. Moreover, the power of an association study based on cases with a family history of the disease is at least twice higher compared to a study using unselected cases [18]. As data of well-known risk factors (age of menarche, history of pregnancy etc.) were not available, we ignored to test for gene-environment interaction.

## Conclusion

Both the BRIP1 -64G>A and Pro919Ser variants show no effect on familial BC risk in the German population.

## Abbreviations

BC – breast cancer

BACH1 – BRCA1-associated C-terminal helicase

BRIP1 – BRCA1-interacting protein 1

DHPLC – denaturing high performance liquid chromatography

95% C.I. – 95% confidence interval

OR – odds ratio

SNP – single nucleotide polymorphism

## Competing interests

The author(s) declare that they have no competing interests.

## Authors' contributions

BF conducted the experiments, performed data acquisition and interpretation, and drafted the manuscript. KH participated in the study coordination and revised the manuscript. AM, BW, CS, MK, PB, RKS and CRB collected DNA samples and were responsible for the *BRCA1/BRCA2 *mutation screening. BB designed and coordinated the study and reviewed the manuscript. All authors read and approved the final version of the submitted manuscript.

## Pre-publication history

The pre-publication history for this paper can be accessed here:



## Figures and Tables

**Table 1:** Genotype distributions of the BRIP1 variants -64G>A (rs2048718^§^) and Pro919Ser (rs4986764^§^) among unrelated German *BRCA1/2 *mutation-negative familial breast cancer patients and healthy, unrelated female control subjects

**BRIP -64G>A**	**CASES**		**CONTROLS**				
	**n**	**%**	**n**	**%**	OR*	**95% CI**	***P***
**All **Σ	**571**		**712**				

GG	181	31.7	228	32.0	1		
GA	283	49.6	340	47.8	1.05	0.82–1.35	0.71
AA	107	18.7	144	20.2	0.94	0.68–1.29	0.68
GA+AA	390	68.3	484	68.0	1.02	0.80–1.29	0.90

**< 50 years **Σ	**406**		**367**				

GG	132	32.5	113	30.8	1		
GA	190	46.8	180	49.0	0.90	0.65–1.25	0.54
AA	84	20.7	74	20.2	0.97	0.65–1.45	0.89
GA+AA	274	67.5	254	69.2	0.92	0.68–1.25	0.61

**50 years **Σ	**165**		**345**				

GG	49	29.7	115	33.3	1		
GA	93	56.4	160	46.4	1.36	0.90–2.08	0.15
AA	23	13.9	70	20.3	0.77	0.43–1.37	0.38
GA+AA	116	70.3	230	66.7	1.18	0.79–1.77	0.41

**BRIP Pro919Ser**							

**All **Σ	**571**		**712**				

Pro/Pro	181	31.7	226	31.7	1		
Pro/Ser	295	51.7	365	51.3	1.01	0.79–1.29	0.94
Ser/Ser	95	16.6	121	17.0	0.98	0.70–1.37	0.91
Pro/Ser+Ser/Ser	390	68.3	486	68.3	1.00	0.79–1.27	0.97

**< 50 years **Σ	**406**		**367**				

Pro/Pro	121	29.8	118	32.2	1		
Pro/Ser	219	53.9	182	49.6	1.17	0.85–1.62	0.33
Ser/Ser	66	16.3	67	18.3	0.96	0.63–1.47	0.85
Pro/Ser+Ser/Ser	285	70.2	249	67.8	1.12	0.82–1.51	0.48

**50 years **Σ	**165**		**345**				

Pro/Pro	60	36.4	108	31.3	1		
Pro/Ser	76	46.1	183	53.0	0.75	0.49–1.13	0.17
Ser/Ser	29	17.6	54	15.7	0.97	0.56–1.68	0.90
Pro/Ser+Ser/Ser	105	63.6	237	68.7	0.80	0.54–1.18	0.26

*As compared to GG and Pro/Pro homozygotes, respectively; § dbSNP rs#; Hardy-Weinberg equilibrium test was undertaken using Pearson's goodness-of-fit chi-square test with one degree of freedom. Adjustment for age did not change the ORs, assuming that the distribution of the BRIP1 genotypes is age-independent.
